# Urate-lowering therapy for gout and asymptomatic hyperuricemia in the pediatric population: a cross-sectional study of a Japanese health insurance database

**DOI:** 10.1186/s12887-021-03051-x

**Published:** 2021-12-18

**Authors:** Masataka Honda, Hideki Horiuchi, Tomoko Torii, Akihiro Nakajima, Takeshi Iijima, Hiroshi Murano, Hisashi Yamanaka, Shuichi Ito

**Affiliations:** 1 Clinical Research Support Center, Tokyo Metropolitan Children’s Medical Center, Musashidai 2-8-29, Fuchu-shi, Tokyo 183-8561 Japan; 2 Medical Science Department, Teijin Pharma Limited, Kasumigaseki Common Gate West Tower, Kasumigaseki 3-2-1, Chiyoda-ku, Tokyo 100-8585 Japan; 3 Pharmaceutical Development Administration Department, Teijin Pharma Limited, Kasumigaseki Common Gate West Tower, Kasumigaseki 3-2-1, Chiyoda-ku, Tokyo 100-8585 Japan; 4 Pharmaceutical Development Coordination Department, Teijin Pharma Limited, Kasumigaseki Common Gate West Tower, Kasumigaseki 3-2-1, Chiyoda-ku, Tokyo 100-8585 Japan; 5 Rheumatology, Sanno Medical Center, Akasaka 8-5-35, Minato-ku, Tokyo 107-0052 Japan; 6 Department of Rheumatology, International University of Health and Welfare, Kozunomori 4-3, Narita-shi, Chiba 286-8686 Japan; 7 Institute of Rheumatology, Tokyo Women’s Medical University, Kawada-cho 8-1, Shinjuku-ku, Tokyo 162-8666 Japan; 8 Department of Pediatrics, Graduate School of Medicine, Yokohama City University, Fukuura 3-9, Kanazawa-ku, Yokohama-shi, Kanagawa 236-0004 Japan

**Keywords:** Database, Epidemiology, Gout, Hyperuricemia, Children, Hyperuricemia drug therapy

## Abstract

**Background:**

Our previous research showed that uric acid lowering therapy (ULT) for gout and hyperuricemia is being prescribed for pediatric patients even though these drugs have not been approved for use in children. However, the actual clinical situation has not been clearly elucidated. In this paper, we provide an in-depth look at the details of actual clinical practice.

**Methods:**

This retrospective cross-sectional study accessed health insurance data for 696,277 children from April 2016 through March 2017 to identify pediatric patients with gout or asymptomatic hyperuricemia, calculate the proportion of patients prescribed ULTs, and analyze population characteristics. Adherence and mean dose for febuxostat and allopurinol, the most commonly prescribed drugs, were also analyzed.

**Results:**

Among children with gout or asymptomatic hyperuricemia, we found that 35.1% (97/276) were prescribed ULT. This proportion increased with age, especially among males. By comorbidity, ULT was prescribed to 47.9% (46/96) of patients with kidney disease, 41.3% (26/63) for cardiovascular disease, 40.0% (6/15) for Down syndrome, and 27.1% (32/118) for metabolic syndrome. In patients with kidney disease, febuxostat was prescribed more than twice as frequently as allopurinol (28 vs. 12). Median values for the medication possession ratio (MPR) of febuxostat and allopurinol were 70.1 and 76.7%, respectively, and prescriptions were continued for a relatively long period for both drugs. Both drugs were prescribed at about half the adult dose for patients 6–11 years old and about the same as the adult dose for patients 12–18 years old.

**Conclusions:**

This study showed that the continuous management of serum uric acid is being explored using off-label use of ULT in pediatric patients with gout or asymptomatic hyperuricemia in Japan. Drug selection is based on patient characteristics such as sex, age, and comorbidities, and pediatric dosage is based on usage experience in adults. To develop appropriate pediatric ULT, clinical trials are needed on the efficacy and safety of ULT in the pediatric population.

**Trial registration:**

UMIN000036029.

**Supplementary Information:**

The online version contains supplementary material available at 10.1186/s12887-021-03051-x.

## Background

Gout and asymptomatic hyperuricemia are treated to reduce the painful symptoms of gouty arthritis and gout tophi, and also to prevent the development of gout-related kidney disease and uric acid kidney stones. In pediatric patients with persistent hyperuricemia, dual-energy computerized tomography scans show urate crystal deposition very similar to that seen in adults with persistent hyperuricemia [1–3]. However, the causes of hyperuricemia in adult patients are more often related to lifestyle, while hyperuricemia in pediatric patients is usually due to underlying chronic disease, including cardiovascular and kidney disease, and to inborn errors of metabolism [4–15].

In a previous study, we accessed a Japanese health insurance database to study the incidence and prevalence of gout and of asymptomatic hyperuricemia in children and to investigate patient characteristics in that population [16]. To the best of our knowledge, that report was the first description of pediatric gout and asymptomatic hyperuricemia in a real-world clinical setting. Our findings showed that a certain number of pediatric patients had gout or asymptomatic hyperuricemia and that the prevalence of these conditions increased with age. In particular, many pediatric patients with gout or asymptomatic hyperuricemia also had cardiovascular disease and/or kidney disease. After puberty, we also noted an increase in the number of patients with comorbid metabolic syndrome associated with lifestyle-related diseases [16].

We also found that urate-lowering therapy (ULT) was frequently prescribed for pediatric patients with gout or asymptomatic hyperuricemia, even though those drugs have not yet been approved for pediatric indications in Japan [16]. In adult gout patients, ULTs are typically prescribed to prevent the development of gouty arthritis by reducing serum uric acid levels to < 6.0 mg/dL [17–21]. In general, pediatric studies tend to have markedly fewer patients than studies in adults, and each drug formulation and dosage must be investigated in each age group within the pediatric population. These factors, which contribute to delays in drug development, also apply to gout and hyperuricemia in pediatric patients. As a result, ULT is not yet internationally approved for gout or hyperuricemia in children, and there is almost no mention of pediatric gout or hyperuricemia in treatment guidelines in Europe and the United States [17, 18]. There are thus very few reports of ULT to treat hyperuricemia in pediatric patients with gout, and the real-world status of such treatment has not been clearly documented.

Unlike Europe and the United States, Japan considers the risk of gouty arthritis in hyperuricemic individuals, and ULT is recommended for adults with asymptomatic hyperuricemia who do not have a history of gout but do have serum uric acid ≥8.0 mg/dL and renal or cardiovascular comorbidities, as well as for otherwise healthy patients who have serum uric acid ≥9.0 mg/dL [19, 20]. However, almost no research has been published on how these adult treatment conditions affect therapeutic interventions in hyperuricemic children, particularly those with asymptomatic hyperuricemia.

The present cross-sectional study builds on our previous work [16], using data gathered from medical database records of patients who were covered by Japanese health insurance. Our objective was to examine the real-world clinical treatment of pediatric patients with gout or asymptomatic hyperuricemia in Japan. This type of information is needed to determine what kinds of treatments are appropriate for gout and asymptomatic hyperuricemia in children.

## Methods

Most of the methodology of this study has been reported previously [16] and is summarized here.

### Study design

This retrospective study accessed a health insurance database for data from April 2016 through March 2017. We used the data to investigate the prevalence of gout and asymptomatic hyperuricemia in the Japanese pediatric population during that period and to assess patient characteristics and treatment conditions. The study was registered in the UMIN Clinical Trials Registry (2019/2/27, UMIN000036029). The health insurance database was the JMDC Claims Database, one of the largest insurance claims databases in Japan, which contained information on approximately 2% of the Japanese population at the time of this study [22]. Age distribution of pediatric members was similar to that of the overall Japanese pediatric population for the period 2016 to 2017. The database consists of anonymized information from multiple health insurance organizations that serve corporate-employed members and their families and that include diagnosis codes and drug prescription records. JMDC has permission to share some of that information with third parties for research. Patients are assigned identification numbers so their data can be traced through multiple hospitals and clinical facilities.

### Participants

Patients were eligible for inclusion in this study if they were continuously registered in the JMDC Claims Database from April 2016 to March 2017, had been diagnosed with gout or asymptomatic hyperuricemia, and were 0 to 18 years of age as of April 2016. To exclude cases of chemotherapy-induced hyperuricemia, patients with malignant tumors were removed from consideration. “Target population” as used in this study is defined in Supplemental Appendix 1 (Table S1).

### Study measures

We identified four points based on data from April 2016 to March 2017. First was the proportion of patients who received ULT for gout or asymptomatic hyperuricemia. Second was the characteristics of those patients, focusing on the four comorbidities of kidney disease, cardiovascular disease, metabolic syndrome, and Down syndrome. Except for Down syndrome, these are common comorbidities of hyperuricemia for which ULT is recommended in adult treatment guidelines in Japan [19, 20], and Down syndrome is often associated with hyperuricemia in pediatric patients [11, 12]. Third was the number of patients who were prescribed each ULT. The drugs that are indicated for gout and hyperuricemia and were available in Japan during the study period included the xanthine oxidase inhibitors allopurinol, febuxostat [23], and topiroxostat [24], and the uricosuric agents benzbromarone and probenecid. Fourth was ULT adherence and mean dose. For this study, we expected that ULTs would be prescribed to only a few pediatric patients, so we limited our analysis to febuxostat and allopurinol, which are the most commonly prescribed ULTs in adult patients [25].

The definitions of “comorbidity” and “ULT” are provided in Supplemental Appendix 1 (Table S1).

### Statistical methods

Sample size was not calculated because this was a cross-sectional study to investigate actual clinical situations. All insurance subscribers who met the eligibility criteria were included. The proportion of ULT prescriptions was then calculated, using the number of patients prescribed ULT as the numerator and the number of gout or asymptomatic hyperuricemia patients as the denominator. Data were calculated for all patients, males, and females, and for each age subgroup and each disease state subgroup (gout or asymptomatic hyperuricemia). The proportion of ULT prescriptions was calculated for each comorbidity in all patients and in each age subgroup. Patient characteristics (sex, comorbidities, and prescribed drugs) for the patients prescribed ULT were summarized overall and by age group.

We also calculated the number of patients prescribed each ULT drug for all patients and for subgroups of age, sex, and comorbidity. Double counting was allowed for those patients who were prescribed multiple drugs during the study period. We then calculated the number of days ULT was prescribed and the medication possession ratio (MPR; the ratio of the number of days ULT was prescribed to the 365 days from April 1, 2016 to March 31, 2017 during which the patient was eligible for that prescription) for febuxostat and allopurinol. Mean prescription doses were calculated for all patients and for each age group and were expressed as the mean and median based on the annual mean of prescriptions for each patient in the group.

For normally distributed data, mean and standard deviation (SD) were calculated. For non-normally distributed data, median and interquartile range (IQR) were calculated. Categorical variables were summarized by number and proportion of patients. All analyses and data processing were performed using SAS version 9.4.

## Results

### Study population

**Fig. 1:**
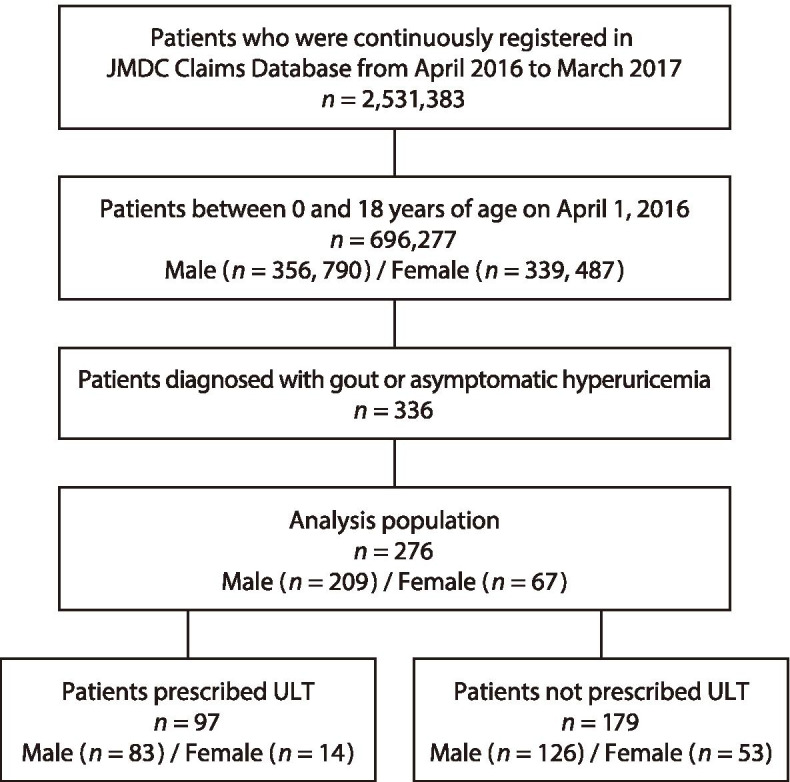
Disposition of patients in the analysis set. ULT: urate-lowering therapy

As previously reported [16], the study population totaled 696,277 children aged 0 to 18 years (as of April 1, 2016), based on the number of pediatric insurance subscribers who were registered in the JMDC Claim Database without interruption from April 2016 to March 2017. For this study, 276 patients identified as having gout or asymptomatic hyperuricemia were included in analysis (Fig. 1).

### Proportion of patients diagnosed with gout or asymptomatic hyperuricemia who received ULT

Overall, ULT was prescribed for 35.1% (97/276) and that number trended higher with age [16]. ULT was prescribed for 37.5% (18/48) with gout and 34.6% (79/228) with asymptomatic hyperuricemia. Details are shown by sex and by age in Table 1. Only one male and no females received ULT in the group 0–5 years of age, but the proportion of ULT prescriptions increased with age, particularly in males.

**Table 1:** Number and proportion of patients diagnosed with gout or asymptomatic hyperuricemia who received ULT

		Age		
	Total	0–5 years	6–11 years	12–18 years
All				
All				
Diagnosed, *n*	276	22	43	211
Treated, *n* (%)	97 (35.1)	1 (4.5)	10 (23.3)	86 (40.8)
Gout				
Diagnosed, *n*	48	3	7	38
Treated, *n* (%)	18 (37.5)	0 (0.0)	1 (14.3)	17 (44.7)
Asymptomatic hyperuricemia				
Diagnosed, *n*	228	19	36	173
Treated, *n* (%)	79 (34.6)	1 (5.3)	9 (25.0)	69 (39.9)
Male				
All				
Diagnosed, *n*	209	10	23	176
Treated, *n* (%)	83 (39.7)	1 (10.0)	6 (26.1)	76 (43.2)
Gout				
Diagnosed, *n*	33	1	2	30
Treated, *n* (%)	16 (48.5)	0 (0.0)	0 (0.0)	16 (53.3)
Asymptomatic hyperuricemia				
Diagnosed, *n*	176	9	21	146
Treated, *n* (%)	67 (38.1)	1 (11.1)	6 (28.6)	60 (41.1)
Female				
All				
Diagnosed, *n*	67	12	20	35
Treated, *n* (%)	14 (20.9)	0 (0.0)	4 (20.0)	10 (28.6)
Gout				
Diagnosed, *n*	15	2	5	8
Treated, *n* (%)	2 (13.3)	0 (0.0)	1 (20.0)	1 (12.5)
Asymptomatic hyperuricemia				
Diagnosed, *n*	52	10	15	27
Treated, *n* (%)	12 (23.1)	0 (0.0)	3 (20.0)	9 (33.3)

*ULT* urate-lowering therapy

### Proportion of ULT treatment, classified by comorbidity, in patients diagnosed with gout or asymptomatic hyperuricemia

Among the 276 patients diagnosed with gout or asymptomatic hyperuricemia, 96 (34.8%) had kidney disease, 63 (22.8%) had cardiovascular disease, 118 (42.8%) had metabolic syndrome, and 15 (5.4%) had Down syndrome [16].

**Fig. 2:**
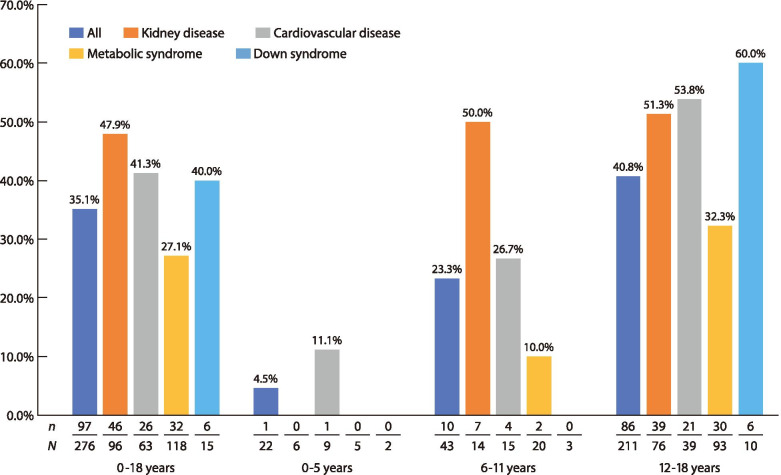
Proportion of patients diagnosed with gout or asymptomatic hyperuricemia who received ULT, by comorbidity. ULT: urate-lowering therapy. *N:* number of patients diagnosed with gout or asymptomatic hyperuricemia; *n:* number of patients diagnosed with gout or asymptomatic hyperuricemia who received ULT

The proportion of ULT prescriptions in relation to each comorbidity is shown in Fig. 2. ULT was prescribed for 47.9% of patients (46/96) with kidney disease, 41.3% (26/63) with cardiovascular disease, 27.1% (32/118) with metabolic syndrome, and 40.0% (6/15) with Down syndrome. The proportion of ULT prescriptions increased with patient age and in the 12–18 group, ULT was prescribed to 51.3% (39/76) of patients with kidney disease and 53.8% (21/39) of patients with cardiovascular disease. The largest subgroup within that age range consisted of 93 patients with metabolic syndrome, of whom 32.3% (30/93) received ULT. This age group included 10 Down syndrome patients, of whom 60% (6/10) received prescriptions for ULT.

### Characteristics of patients treated with ULT

Characteristics of the patients treated with ULT are shown in Table 2. The totals exceed total patient number because some patients had multiple comorbidities. ULT was prescribed to 97 children (83 males and 14 females). Males made up 85.6% of the ULT group and 70.4% of the non-ULT group. In the overall study population, the most common comorbidity was metabolic syndrome [16]. In the subpopulation of patients prescribed ULT, the most common comorbidity was kidney disease (47.4%, 46/97), followed by metabolic syndrome (33.0%, 32/97). Angiotensin-converting enzyme inhibitors (ACEIs) or angiotensin II receptor blockers (ARBs) were used by 24.7% (24/97) of the patients prescribed ULT and by 4.5% (8/179) of the patients not prescribed ULT.

**Table 2:** Characteristics of patients diagnosed with gout or asymptomatic hyperuricemia

	Patients with ULT treatment				Patients with no ULT treatment	All
		Age				
	Total (*n* = 97)	0–5 years (*n* = 1)	6–11 years (*n* = 10)	12–18 years (*n* = 86)	Total (*n* = 179)	Total (*n* = 276)
Sex, *n* (%)						
Male	83 (85.6)	1 (100.0)	6 (60.0)	76 (88.4)	126 (70.4)	209 (75.7)
Female	14 (14.4)	0 (0.0)	4 (40.0)	10 (11.6)	53 (29.6)	67 (24.3)
Comorbidity, *n* (%)						
Kidney disease	46 (47.4)	0 (0.0)	7 (70.0)	39 (45.3)	50 (27.9)	96 (34.8)
Cardiovascular disease	26 (26.8)	1 (100.0)	4 (40.0)	21 (24.4)	37 (20.7)	63 (22.8)
Metabolic syndrome	32 (33.0)	0 (0.0)	2 (20.0)	30 (34.9)	86 (48.0)	118 (42.8)
Down syndrome	6 (6.2)	0 (0.0)	0 (0.0)	6 (7.0)	9 (5.0)	15 (5.4)
Treatment and drug use, *n* (%)						
Dialysis	2 (2.1)	0 (0.0)	0 (0.0)	2 (2.3)	0 (0.0)	2 (0.7)
Cardiovascular disease drug	5 (5.2)	0 (0.0)	4 (40.0)	1 (1.2)	4 (2.2)	9 (3.3)
Diuretic drug	8 (8.2)	1 (100.0)	3 (30.0)	4 (4.7)	5 (2.8)	13 (4.7)
β blocker	5 (5.2)	0 (0.0)	2 (20.0)	3 (3.5)	2 (1.1)	7 (2.5)
Ca antagonist	6 (6.2)	0 (0.0)	1 (10.0)	5 (5.8)	2 (1.1)	8 (2.9)
ACEI and/or ARB	24 (24.7)	1 (100.0)	3 (30.0)	20 (23.3)	8 (4.5)	32 (11.6)
Antihyperlipidemic drug	7 (7.2)	0 (0.0)	0 (0.0)	7 (8.1)	7 (3.9)	14 (5.1)
Antidiabetic drug	1 (1.0)	0 (0.0)	0 (0.0)	1 (1.2)	4 (2.2)	5 (1.8)
Immunosuppressant	9 (9.3)	0 (0.0)	5 (50.0)	4 (4.7)	9 (5.0)	18 (6.5)
Vitamin D	7 (7.2)	0 (0.0)	2 (20.0)	5 (5.8)	6 (3.4)	13 (4.7)

*ULT* urate-lowering therapy, *ACEI* angiotensin-converting enzyme inhibitor, *ARB* angiotensin II receptor blocker

### Number and proportion of patients prescribed various ULTs

Overall, 97 patients were prescribed ULTs. Febuxostat was prescribed to 54 patients, allopurinol 31, benzbromarone 15, topiroxostat 6, and probenecid 1, with totals exceeding the total patient number because some patients received prescriptions for multiple ULTs (Table 3). In patients with kidney disease, febuxostat was prescribed more than twice as frequently as allopurinol (28 vs. 12).

**Table 3:** Number of patients prescribed various ULTs

	Febuxostat	Allopurinol	Benzbromarone	Topiroxostat	Probenecid
Age, *n*					
Total	54	31	15	6	1
0–5 years	0	1	0	0	0
6–11 years	6	3	1	0	0
12–18 years	48	27	14	6	1
Sex, *n*					
Male	48	27	11	6	1
Female	6	4	4	0	0
Comorbidity, *n*					
Kidney disease	28	12	7	4	0
Cardiovascular disease	15	10	2	0	1
Metabolic syndrome	18	14	6	3	1
Down syndrome	4	1	1	0	0

*ULT* urate-lowering therapy

### Number of days of prescription, medication possession ratio, and mean prescribed dose for ULTs (febuxostat and allopurinol)

Febuxostat was prescribed for a median of 256.0 days (IQR 118.0, 343.0) and allopurinol for 280.0 days (IQR 118.0, 360.0) (Table 4). Median values for the MPR were 70.1 (IQR 32.3, 94.0) and 76.7 (IQR 32.3, 98.6), respectively. Overall, the mean prescribed dose±SD in the study population was 15.0 ± 10.2 mg for febuxostat and 126.0 ± 61.8 mg for allopurinol. In the 6–11 year group, 6 patients were prescribed febuxostat and the mean dose was 6.7 ± 2.6 mg. In that group, 3 patients were prescribed allopurinol, and the mean dose was 74.3 ± 28.3 mg. In the 12–18 year group, the mean dose for febuxostat was about 16.0 ± 10.3 mg and for allopurinol, the mean was about 135.6 ± 59.1 mg.

**Table 4:** Number of days of prescription, MPR, and mean prescribed dose of ULTs (febuxostat and allopurinol)

	Febuxostat (*n* = 54)	Allopurinol (*n* = 31)
Prescription, days/year		
Median (Q1, Q3)	256.0 (118.0, 343.0)	280.0 (118.0, 360.0)
MPR		
Median (Q1, Q3)	70.1 (32.3, 94.0)	76.7 (32.3, 98.6)
Dose, mg		
Total (0–18 years)		
Mean (SD)	15.0 (10.2)	126.0 (61.8)
Median (Q1, Q3)	10.0 (10.0, 20.0)	100.0 (100.0, 200.0)
0–5 years ^a^		
Mean	–	18.1
Median	–	18.1
6–11 years ^b^		
Mean (SD)	6.7 (2.6)	74.7 (28.3)
Median (Q1, Q3)	5.5 (5.0, 9.9)	80.0 (44.2, 100.0)
12–18 years ^c^		
Mean (SD)	16.0 (10.3)	135.6 (59.1)
Median (Q1, Q3)	10.0 (10.0, 20.0)	100.0 (100.0, 200.0)

*ULT* urate-lowering therapy, *MPR* medication possession ratio, *Q* quartile, *SD* standard deviation

^a^ Febuxostat (*n* = 0); Allopurinol (*n* = 1), ^b^ Febuxostat (*n* = 6); Allopurinol (*n* = 3),

^c^ Febuxostat (*n* = 48); Allopurinol (*n* = 27)

## Discussion

In this study we investigated the real-world use of ULT for gout and asymptomatic hyperuricemia in children. Even at low prescription levels, we found that the ULT was selected after considering patient characteristics including patient’s comorbidities, that dosage and administration were based on the experience of real-world usage in adults, and that continuous serum uric acid management was tried in pediatric patients with gout or hyperuricemia in much the same way as in adults.

Japan has a universal health insurance system to which all Japanese residents subscribe for comprehensive health insurance through their employer or an administrative agency. The cost of covered medical care is the same across the country, the co-pay for the patient is usually 30% of the total medical cost, and most local governments also offer free or discounted health care to children. Every citizen can be seen at any medical facility at any time, and various biochemical tests are performed during the process of diagnosis at those facilities. In these tests, which are covered by health insurance, the standard test panel usually includes uric acid. Thus, the Japanese healthcare system provides an environment in which hyperuricemia can be detected at a high rate and ULTs are easy to prescribe for those patients.

In the present study, we found that the highest proportion of ULT was prescribed to patients who had kidney or cardiovascular disease. In these patients with hyperuricemia and cardiovascular disease, we found a notable age-related increase in ULT prescriptions. In the kidney disease subgroup, the proportion of ULT use was particularly high in patients aged 6–11 and 12–18. Because the kidneys excrete uric acid, serum uric acid elevation is generally expected in patients with kidney disease, whether children or adults, and some papers have been published on the relationship between kidney disease and serum uric acid level [14, 26, 27]. Our study confirmed that pediatric patients with hyperuricemia and kidney disease were prescribed ULT, and our results were consistent with previous reports. ACEI/ARBs were used by 24.7% of the patients prescribed ULT, in comparison to 4.5% of patients not prescribed ULT. ACEI/ARBs are often prescribed for kidney or cardiovascular disease [28, 29], and these conditions may cause serum uric acid levels high enough to require treatment. However, it remains unclear whether ACEI/ARBs elevate serum uric acid levels.

The subgroup with underlying metabolic syndrome contained the highest number of diagnosed patients, but the proportion receiving ULT was lower than for other comorbidities. The lower level of ULT in those patients may have been because this syndrome is treated primarily by lifestyle changes, and ULT is generally prescribed only if lifestyle changes are insufficient to manage the patient’s condition.

In the Down syndrome group, 40.0% (6/15) received ULT. All 6 were 12–18 years of age, and 5 had multiple comorbidities of kidney disease, cardiovascular disease, or metabolic syndrome. Particular caution is required from puberty, at which time Down syndrome patients begin to experience increasingly high risk of hyperuricemia [12], in part because the comorbidities of lifestyle-related diseases and obesity predispose to uric acid elevation. There have been some cases in which juvenile gouty arthritis occurred in patients with Down Syndrome [11], and that background may also be related to these comorbidities.

Our study showed that febuxostat and allopurinol were the most commonly prescribed ULTs in pediatric patients. In children, MPRs for these drugs were similar to the proportion reported in adults [25]. These findings indicated that ULT was being prescribed for continuous serum uric acid management in pediatric patients in much the same way as in adults. Analysis of mean prescribed doses of febuxostat and allopurinol in pediatric patients showed that approximately half the mean prescribed dose for adults was used in patients aged 6–11 and the same amount in patients aged 12–18 [25]. This may be because the dosage for children was based on that for adults, since dosage and administration have not been established for pediatric use. The number of patients on febuxostat was highest in the kidney comorbidities subgroup, possibly because allopurinol is eliminated by the kidneys [30], while febuxostat has both hepatic and renal elimination routes [31].

In pediatric patients, chronic hyperuricemia is often associated with underlying conditions, such as cardiovascular or renal diseases, inborn errors of purine metabolism, genetic disorders, or kidney transplantation [4–15]. In contrast, hyperuricemia in adult patients is often associated with sexual differences or lifestyle-related diseases. Serum uric acid levels are known to increase with age in male children because of the effects of increased testosterone levels [4], and our previous study showed both age-based and sex-based differences in the prevalence of hyperuricemia [16]. We also found a relationship between age and metabolic syndrome [16], suggesting that adolescent hyperuricemia may be associated with lifestyle-related diseases [32, 33]. Based on the findings above, we hypothesized that the prevalence of hyperuricemia and gout increase because of overlapping factors or effects of changes in age or lifestyle during the transition from childhood to adulthood.

Particularly in patients with underlying conditions as described above, such as inborn errors of metabolism, congenital heart disease, and congenital kidney disease, chronic hyperuricemia can develop at a young age and may result in gout before the child reaches 10 years of age. This situation places an enormous burden on patients and their families. There are several case reports of pediatric gout patients who had familial juvenile hyperuricemic nephropathy or other comorbidities and whose gout became intractable in their 20s or 30s, resulting in joint deformity or tophus [8–10, 34, 35]. Recent years have seen increasing concern about the incidence of gout and asymptomatic hyperuricemia in children, and the potential onset of gouty arthritis at younger ages, as a result of obesity [32, 33]. Previously, we reported that the risk of gouty arthritis is similar in pediatric and adult gout patients [16] and those findings emphasize the potential importance of drug therapy for pediatric patients with gout or asymptomatic hyperuricemia in whom lifestyle modification has been insufficiently effective. In such cases, ULT might prevent the deposition of urate crystals and progression to refractory disease that could occur if hyperuricemia remains untreated into adulthood [36, 37].

Because this was a database study, we were unable to investigate serum uric acid levels at the start of therapeutic intervention and after treatment, or to collect data on safety. In the near future we hope that clinical studies will be performed in pediatric patients to determine the relationship between dosage and uric acid level, to investigate the safety and tolerability of ULTs, and to establish effective drug therapy for gout and asymptomatic hyperuricemia in this population.

This study has several limitations. First, we analyzed data based on information from insurance claims (medical fee claim forms), which were not collected for research purposes, so the validity of definitions for various diseases was not assured. Second, the overall study population was less than 300 patients, and several subgroups contained only a few patients, which limits the generalizability of our findings. Third, data used were from company employees and their family members, which excluded members of other populations, reducing our findings’ generalizability. Fourth, some patients had claims reimbursed through a bundled payment plan, and only limited information may have been available on prescriptions for those patients. Fifth, this was a cross-sectional study with evaluation during a single period, so it was impossible to estimate the causal relationship between exposure and outcome.

## Conclusions

Our study analyzed the use of ULTs in pediatric patients with gout or hyperuricemia, based on data from a Japanese health insurance claims database. Results show that the off-label use of ULTs is being explored in these patients, with drug selection based on patient characteristics and dosage based on usage experience in adults. In pediatric patients, serum uric acid management tends to be continuous, and pediatric prescriptions for ULT tend to increase with patient age, particularly in male patients and those with kidney disease. Clinical trials are needed in the near future on the efficacy and safety of ULTs in the pediatric population to establish appropriate pediatric drug therapy.

## Supplementary Information

**Additional file 1: Table S1** List of definitions.

## Data Availability

The data that support the findings of this study are available from JMDC, but restrictions apply to the availability of these data, which were used under license for the current study, and so are not publicly available. Data are however available from the authors upon reasonable request and with permission of JMDC.

## Abbreviations

ACEI: Angiotensin-converting enzyme inhibitor
ARB: Angiotensin II receptor blocker
IQR: Interquartile range
MPR: Medication possession ratio
SD: Standard deviation
ULT: Urate-lowering therapy
