# Supplementary material for: Urate-lowering therapy for gout and asymptomatic hyperuricemia in the pediatric population: a cross-sectional study of a Japanese health insurance database
Source: BMC Pediatr. 2021 Dec 18;21:581. doi: 10.1186/s12887-021-03051-x (PMC8684120; doi:10.1186/s12887-021-03051-x)
Supplement: Supplementary file 1 — Additional file 1: Table S1 List of definitions. [file 12887_2021_3051_MOESM1_ESM.pdf]

Additional file 1

Urate-lowering therapy for gout and asymptomatic hyperuricemia in the pediatric population: a cross-sectional study of a Japanese health insurance database

Contents:

Table S1 List of definitions **2**

Table S1 List of definitions

Gout

Patients for whom any of the following applied for the period from April 2016 to March 2017.

- Code M10 (Gout) under the ICD10 coding system twice or more in separate months
- Code M10 (Gout) under the ICD10 coding system once or more, and one or more prescriptions for M04 (Antigout preparations) by ATC code and gout diagnosis shown on the same insurance claim form.
- Code M10 (Gout) under the ICD10 coding system once or more, and one or more prescriptions for M01A1 (Anti-rheumatics, non-steroidal plain) or H02A2 (Oral corticosteroids, plain) by ATC code and gout diagnosis shown on the same insurance claim form.

Satisfying the above criteria and with no instances of Code M352 (Behçet's disease) under the ICD10 coding system or Code 8831283 (familial Mediterranean fever) under Standard disease code .

Asymptomatic hyperuricemia

Patients for whom the definition of gout did not apply and any of the following applied for the period from April 2016 to March 2017.

- Code E790 (Hyperuricaemia without signs of inflammatory arthritis and tophaceous disease) under the ICD10 coding system twice or more in separate months.
- Code E790 (Hyperuricaemia without signs of inflammatory arthritis and tophaceous disease) under the ICD10 coding system once or more, and one or more prescriptions for M04 (Antigout preparations) by ATC code and diagnosis for hyperuricaemia without signs of inflammatory arthritis and tophaceous disease shown on the same insurance claim form.

Drug treatment with uric acid lowering drug for gout patients

Patients for whom the following applied for the period from April 2016 to March 2017.

- Satisfying the definition of gout, with code M10 (Gout) under the ICD10 coding system once or more, and one or more prescriptions for M04 (Antigout preparations excluding colchicine) by ATC code and gout diagnosis shown on the same insurance claim form.

Drug treatment with uric acid lowering drug for asymptomatic hyperuricemia patients

Patients for whom the definition of drug treatment with uric acid lowering drugs for gout did not apply and any of the following applied for the period from April 2016 to March 2017.

- Satisfying the definition of asymptomatic hyperuricemia, with code E790 (Hyperuricaemia without signs of inflammatory arthritis and tophaceous disease) under the ICD10 coding system once or more, and one or more prescriptions for M04 (Antigout preparations excluding colchicine) by ATC code and diagnosis for hyperuricaemia without signs of inflammatory arthritis and tophaceous disease shown on the same insurance claim form.

Malignant tumor

Patients for whom the following condition applied for the period from April 2016 to March 2017.

- Code C00-C97 (Malignant neoplasm) or D00-D09 (In situ neoplasms) under the ICD10 coding system

Comorbidities

Coded by ICD10 coding system once or more from April 2016 to March 2017.

- Kidney disease: N00-N08 (Glomerular diseases), N10-N16 (Renal tubulo-interstitial diseases), N17-N19 (Renal failure), N20-N23 (Urolithiasis), N25-N29 (Other disorders of kidney and ureter), I12 (Hypertensive renal disease), I15 (Secondary hypertension), Q60-Q64 (Congenital malformations of the urinary system)
- Cardiovascular disease: I05-I09 (Chronic rheumatic heart diseases), I11 (Hypertensive heart disease), I20-I25 (Ischemic heart diseases), I26-I28

(Pulmonary heart disease and diseases of pulmonary circulation), I30-I52 (Other forms of heart disease), I70-I79 (Diseases of arteries, arterioles and capillaries), Q20-Q28 (Congenital malformations of the circulatory system)

- Metabolic syndrome: E10-E14 (Diabetes mellitus), R73 (Elevated blood glucose level), E66 (Obesity), E78 (Disorders of lipoprotein metabolism and other lipidaemias)
- Down syndrome: Q90 (Down’s syndrome)

Treatment

Coded for receipt of treatment once or more from April 2016 to March 2017.

-Dialysis: 114003610 (Additional fee for second or third time guidance <guidance fee for ambulatory peritoneal dialysis>), 114003510 (Guidance fee for ambulatory peritoneal dialysis), 114009310 (Guidance fee for in-home hemodialysis), 114009410 (Additional fee for second or third time guidance <guidance fee for in-home hemodialysis>), 114006610 (Automated peritoneal dialysis system fee), 140029850 (Continuous slow hemofiltration), 140053670 (Additional fee for the disabled etc. <continuous slow hemofiltration>), 140051110 (Artificial kidney, chronic maintenance dialysis [for 5 hours or more]); 140051010 (Artificial kidney, chronic maintenance dialysis [for 4-5 hours]), 140036710 (Artificial kidney, chronic maintenance dialysis [for less than 4 hours]), 140052810 (Artificial kidney, chronic maintenance diafiltration [complicated]), 140007910 (Additional fee for after-hour/holiday <artificial kidney>), 140007710 (Artificial kidney [other]), 140033770 (Additional fee for the disabled etc. <artificial kidney>), 140008170 (Additional fee of induction period <artificial kidney>), 140052570 (Additional fee for ensuring dialysate quality [1] <artificial kidney>), 140052970 (Additional fee for ensuring dialysate quality [2] <artificial kidney>), 140055970 (Additional fee for patient management for lower extremity peripheral arterial disease <artificial kidney>), 140054950 (Additional fee of induction period for 15-30 days, for infant/preschool child <peritoneal dialysis>), 140054850 (Additional fee of induction period [14 days] for infant/preschool child <peritoneal dialysis>), 140008770

(Additional fee of induction period [14 days] <peritoneal dialysis>), 140008670 (Additional fee for abdominal catheter placement <peritoneal dialysis, continuous ambulatory>), 140008510 (Peritoneal dialysis, continuous ambulatory), 140008810 (Peritoneal dialysis [other])

Drugs

- Cardiovascular disease drug: ATC code C01 (Cardiac therapy)
- Diuretic drug: ATC code C03 (Diuretics)
- β blocker: ATC code C07 (beta blocking agents)
- Ca antagonist: ATC code C08 (Calcium antagonists)
- ACE inhibitor and/or ARB: ATC code C09 (Agents acting on the renin-angiotensin system)
- Antihyperlipidemic drug: ATC codes C10 (Lipid regulating/anti-atheroma preparations)
- Antidiabetic drug: ATC code A10 (Drugs used in diabetes)
- Immunosuppressant: ATC code L04X (Other immunosuppressants)
- Vitamin D: ATC code A11C2 (Vitamin D)
- Uric acid lowering drug*: ATC code M04 (Antigout preparations); note that colchicine is excluded

* Use is defined as follows: Code M10 (Gout) or E790 (Hyperuricaemia without signs of inflammatory arthritis and tophaceous disease) under the ICD10 coding system once or more, and one or more prescriptions for each ATC code and diagnosis shown on the same insurance claim form is given during the survey period.

ICD10 International statistical classification of diseases and related health problems 10th revision, *ATC* anatomical therapeutic chemical classification system, *ACE* angiotensin-converting enzyme, *ARB* angiotensin II receptor blocker
